# Complete Genome Sequence of *Streptococcus thermophilus* KLDS 3.1003, A Strain with High Antimicrobial Potential against Foodborne and Vaginal Pathogens

**DOI:** 10.3389/fmicb.2017.01238

**Published:** 2017-07-11

**Authors:** Smith E. Evivie, Bailiang Li, Xiuyun Ding, Yueyue Meng, Shangfu Yu, Jincheng Du, Min Xu, Wan Li, Da Jin, Guicheng Huo, Fei Liu

**Affiliations:** ^1^Key Laboratory of Dairy Science, Ministry of Education, College of Food Sciences, Northeast Agricultural University Harbin, China; ^2^Food Science and Nutrition Unit, Department of Animal Science, Faculty of Agriculture, University of Benin Benin City, Nigeria

**Keywords:** *Streptococcus thermophilus*, genome sequence, EPS, antimicrobial activity, dairy industry

## Abstract

Lactic acid bacteria play increasingly important roles in the food industry. *Streptococcus thermophilus* KLDS 3.1003 strain was isolated from traditional yogurt in Inner Mongolia, China. It has shown high antimicrobial activity against selected foodborne and vaginal pathogens. In this study, we investigated and analyzed its complete genome sequence. The *S. thermophilus* KLDS 3.1003 genome comprise of a 1,899,956 bp chromosome with a G+C content of 38.92%, 1,995 genes, and 6 rRNAs. With the exception of *S. thermophilus* M17TZA496, *S. thermophilus* KLDS 3.1003 has more tRNAs (amino acid coding genes) compared to some *S. thermophilus* strains available on the National Centre for Biotechnology Information database. MG-RAST annotation showed that this strain has 317 subsystems with most genes associated with amino acid and carbohydrate metabolism. This strain also has a unique EPS gene cluster containing 23 genes, and may be a mixed dairy starter culture. This information provides more insight into the molecular basis of its potentials for further applications in the dairy and allied industries.

## Introduction

Lactic acid bacteria (LAB) species are recognized globally as an industrially important group of bacteria used for the production of fermented foods such as yogurt, cheese, and butter (Gezginc et al., 2015; Labrie et al., 2015). While some act as commensals and colonizers on the mucosal surface of our gastrointestinal tracts, others are marketed as probiotics used in foods to improve nutrition and health (LAB, 2011; Enujiugha and Badejo, 2015; Canani et al., 2016; Evivie et al., 2017). As the second most important species of LAB, *Streptococcus thermophilus* has been used for a variety of applications in the dairy and allied industry (Iyer et al., 2010; Kang et al., 2012). While some strains have been shown to produce high amounts of exopolysaccharides (EPS) (Wu et al., 2014; Bai et al., 2016) and bacteriocins (Renye et al., 2016), others have been reported to have a range of probiotic properties which include lowering the effects of diarrhea in young children (Kort et al., 2015), adhesion to intestinal epithelial cells (Kebouchi et al., 2016), anti-inflammatory (Li and Shah, 2016), anti-carcinogenic (Sah et al., 2016), antioxidant (Lee et al., 2015), and bacterial vaginosis-suppressive (Patras et al., 2015) effects.

The need to explore and extensively study microbial strains which have high antimicrobial properties against the spread of notable food pathogens such as bacteria, mold, and yeast can be strategic and novel in the fight to ensure that consumers have safe and nutritious foods. Strains that inhibit the development of vaginal pathogens can also be of high economic value and present new frontiers in the treatment of diverse illnesses (Ankolekar, 2013; Sah et al., 2016). The strain, *S. thermophilus* KLDS 3.1003 has been shown in recent experiments in our laboratory to possess strong antimicrobial activity (expressed as minimum inhibition zones) against pathogenic *Escherichia coli* ATCC25922, *Staphylococcus aureus* ATCC25923 and *Gardnerella vaginalis* ATCC14018 giving 6.40 ± 0.26, 3.43 ± 2.97, and 5.47 ± 0.04 mm, respectively. The cell-free supernatants (CFS) of this strain were also shown to have antagonistic effects against the above-mentioned pathogens, giving 90.42 ± 0.87, 90.97 ± 0.88, and 90.49 ± 0.62% inhibition, respectively, with catalase treatment (data not shown). Here, the complete genome sequence of *S. thermophilus* KLDS 3.1003 is reported to give insight on the molecular basis for its various potential industrial applications in the food industry.

## Methodology and bioinformatics of *S. thermophilus* KLDS 3.1003

*Streptococcus thermophilus* KLDS 3.1003 was isolated from traditional yogurt culture found in Inner Mongolia, China. The whole genome sequencing of *S. thermophilus* KLDS 3.1003 was performed using Pacbio RSII (20K library) and Illumina Hiseq 4000 (500 bp PCR-free library) strategies respectively. Then, 402M Hiseq and 556M Pacbio clean data were generated using a refined data filter. PacBio reads were assembled using the protocol in SMRT Analysis v2.3.0 Pipe: RS_HGAP_Assembly3 following the procedure of Chin et al. (2013) and GATK analysis protocol was used to correct single base errors (Li et al., 2009). The genome sequence of this strain was assembled into a contig of 1,899,956 bp and a total of 38,282 polymerase reads were obtained via Pacbio RSII strategy. The assembly of *S. thermophilus* KLDS 3.1003 was uploaded for annotation using the Metagenomics Rapid Annotation using Subsystem Technology (MG-RAST) (Meyer et al., 2008).

## Results

**Figure 1:**
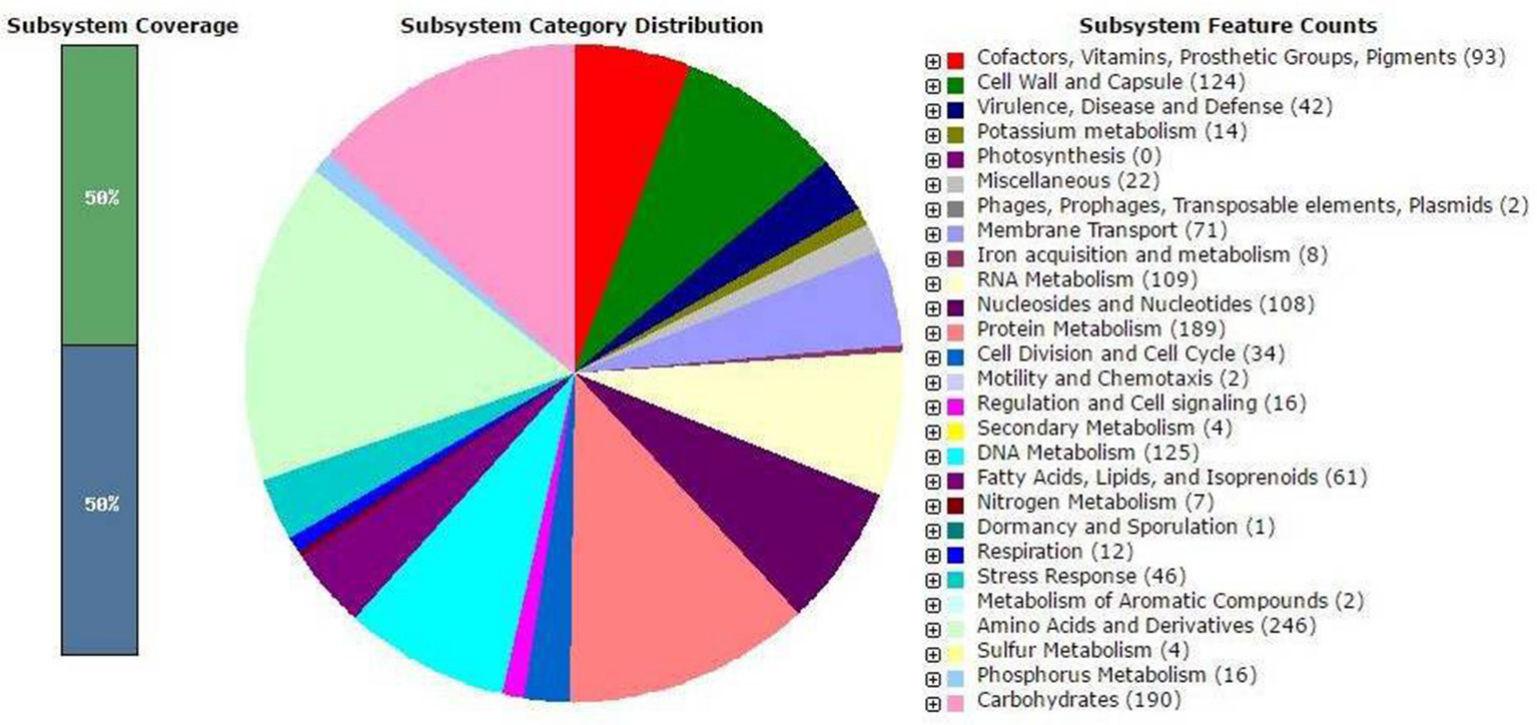
Annotation of *Streptococcus thermophilus* KLDS 3.1003 as generated by the Metagenome Rapid Annotation using Subsystem Technology (MG-RAST).

The complete genome sequence of *S. thermophulus* KLDS 3.1003 was shown to have a G+C content of 38.92%. It has a total of 1,997 genes comprising of 1,731 protein-coding genes, 6 rRNAs, 68 tRNAs, 4 ncRNAs, and 176 pseudo genes (Table 1). These results have also been compared with those of other *S. thermophilus* strains earlier reported such as ASCC 1275 (Wu et al., 2014), MN-BM-A02 (Shi et al., 2015), ND03 (Sun et al., 2011), CNRZ1066, and LMG18311 (Bolotin et al., 2004) (see S1). *Streptococcus thermophilus* KLDS 3.1003 has the highest number of tRNA proteins (total of 68) than all the above-mentioned strains. With the exception of *S. thermophilus* M17TZA496 with a total of 79 tRNA proteins, *S. thermophilus* KLDS 3.1003 has more amino acid-coding genes than all other sequenced *S. thermophilus* genome available on the NCBI database till date. The RAST annotation has assigned the genes of this strain into 317 subsystems with most genes associated with amino acids and derivatives metabolism (15.89%), followed by carbohydrates metabolism (12.27%), and then the protein metabolism subsystems (12.21%). No genes were associated with photosynthetic reactions (see Figure 1).

**Table 1:** General genome features of *Streptococcus thermophilus* KLDS 3.1003.

**Features**	**Chromosome**
Genome size (bp)	1,899,956
Contig numbers	1
G+C content (%)	38.90
Total number of genes	1,997
Number of CDSs	1,731
rRNA genes	6
tRNA genes	68
ncRNAs	4
Pseudo genes	176
GenBank accession no.	CP016877.1

The genome of *S. thermophilus* KLDS 3.1003 has three (3) Comparative Analysis of Clustered Regularly Interspaced Short Palindromic Repeats (CRISPR) loci which were classified as types I-A (CRISPR 1), III-A (CRISPR 2), and II-A (CRSIPR 3), respectively. In addition, KLDS 3.1003 has 11, 14, and 1 spacers in CRISPR 1, CRISPR 2, and CRISPR 3 loci, respectively. The HNH-type system, enshrined in the type II-A CRISPR Cas loci, is rich in restriction enzymes as well as being likely responsible for target cleavage (Kleanthous et al., 1999; Garneau et al., 2010). The CRISPR immune system provides an adaptive defense mechanism against phages and other mobile genetic elements (Mojica et al., 2005; Barrangou et al., 2007). In addition, the CRISPR-cas system is important in assessing the ability of bacteria strains to resist food-borne pathogens (Wu et al., 2014; Li P. et al., 2016; Li W. et al., 2016). Given these properties, the host–phage interactions of this strain as well as the mechanism therein may require further studies.

The *S. thermophilus* KLDS 3.1003 genome has an EPS cluster (from BEN15_RS08500 to BEN15_RS08557 loci) consisting of 23 genes (see S2). In addition to *eps1C* (BEN15_RS08565), *ID* (BEN15_RS08560), *2C* (BEN15_RS08525), and *2D* (BEN15_RS08520), *S. thermophilus* KLDS3.1003 genome also contains *epsA* (BEN15_RS08575), *epsB* (BEN15_RS08570), *epsE* (BEN15_RS08555), *epsF* (BEN15_RS08550), *epsG* (BEN15_RS08540), *epsH* (BEN15_08530), *epsI* (BEN15_08530), eps*J* (BEN15_RS08530), eps*K* (BEN15_RS08515), *epsL* (BEN15_RS08510), *epsM* (BEN15_RS08505), *epsN* (BEN15_RS08500), *epsO* (BEN15_RS0190), *epsP* (BEN15_RS08475), *epsQ* (BEN15_RS00260, BEN15_RS00265), and transposase (BEN15_RS08545). Some of these have been earlier reported to be part of the key enzymes necessary for the polymerization and export of EPS (Jolly and Stingele, 2001). Interestingly, the eps1C-ID and eps2C-2D produced by this organism suggests that it is a mixed producer of capsular and ropy EPS, a rare feature similar to high EPS-producing strains like *S. thermophilus* ASCC 1275 (Wu et al., 2014). This is however subject to further validation as many LABs produce different amounts and types of EPS under different conditions (Caggianiello et al., 2016). Furthermore, eps1C, 1D and eps2C, 2D function in chain length determination and eps A and B facilitate regulation of cell activities (Wu et al., 2014). In comparing the EPS gene cluster of this strain with other prominent EPS-producing strains such as *S. thermophilus* ASCC 1274 (Wu et al., 2014) via BLAST (https://blast.ncbi.nlm.nih.gov/Blast.cgi) we confirm that the genome of both strains are highly similar, possessing unique properties that confer high potentials for *S. thermophilus* KLDS 3.1003 in the dairy industry. Furthermore, *S. thermophilus* KLDS 3.1003 possesses the rare EPS-related enzymes acetyl transferase (BEN15_RS00090) and galactosyl transferase (BEN15_RS08555) which are lacking in all the sequenced *S. thermophilus* strains earlier compared in this report (see S2). Since EPS is known to play important roles in the improvement of texture and viscosity of yogurt and other fermented milks (Duboc and Mollet, 2001), it would thus be important to determine the amount and composition of EPS produced by this strain. In addition, studies aimed to further elucidate the structure and functionalities of its EPS so as to determine specific areas of application are necessary. Investigating the roles of its EPS in low pH tolerance or storage tolerance using isogenic lines will also provide more information in terms of characterization and possible applications in the food and non-food industries.

In all, information on the whole genome sequence of *S. thermophilus* KLDS3.1003 presented here provides the research and industrial community with a deeper dataset of the genetic setup for the potential antimicrobial properties and by extension its potential industrial applications.

## Nucleotide sequence accession number

The complete genome sequence of *S. thermophilus* KLDS 3.1003 has been deposited in GenBank under the accession number CP016877. This strain is also available at Dairy Industrial Culture Collection of the Key Laboratory of Dairy Science (KLDS-DICC), Northeast Agricultural University, Harbin, China.

## Author contributions

GH conceived the project idea. SE was assigned the project and worked with all other authors to successfully extract the genome of *Streptococcus thermophilus* KLDS 3.1003. SE drafted the manuscript. All authors approved the final manuscript draft.

### Conflict of interest statement

The authors declare that the research was conducted in the absence of any commercial or financial relationships that could be construed as a potential conflict of interest.
