# Supplementary material for: Complete Genome Sequence of Streptococcus thermophilus KLDS 3.1003, A Strain with High Antimicrobial Potential against Foodborne and Vaginal Pathogens
Source: Front Microbiol. 2017 Jul 11;8:1238. doi: 10.3389/fmicb.2017.01238 (PMC5504653; doi:10.3389/fmicb.2017.01238)
Supplement: Supplementary file 1 [file Table1.docx]

Supplementary Material

**Complete Genome Sequence of *Streptococcus thermophilus* KLDS 3.1003, a potential probiotic strain with high antimicrobial potential against foodborne and vaginal pathogens**

Smith Etareri Evivie^a,b,1^, Bailiang Li^a,1^, Xiuyun Ding^a^, Yueyue Meng^a^, Shangfu,Yu^a^, Jincheng Du^a^, Min Xu^a^, Wan Li^a^, Da Jin^a^, Guicheng Huo^a*^ and Fei Liu^a*^

^a^ Key Laboratory of Dairy Science, Ministry of Education, College of Food Sciences, Northeast Agricultural University, Harbin 150030, PR China

^b^ Food Science and Nutrition Unit, Department of Animal Science, Faculty of Agriculture, University of Benin, PMB 1154 Benin City, Nigeria

***Correspondence:**Guicheng HUO Ph.D, Professor of Dairy Science

Key Laboratory of Dairy Science, Ministry of Education, College of Food Sciences, Northeast Agricultural University, Harbin 150030, PR China, Tel: +86 451 55191807; Fax: +86 451 55190340

Email address: gchuo58@126.com

^1^These authors contributed equally to this work

S1

| Features | KLDS 3.1003 | ASCC 1275 | ND03 | CNRZ  1066 | MN-BM-A02 | LMG 18311 |
| --- | --- | --- | --- | --- | --- | --- |
| Chromosome size (bp) | 1,899,956 | 1,845,495 | 1,831,949 | 1,796,226 | 1,850,434 | 1,796,846 |
| Contig numbers | 1 | 0 | 0 | 0 | 0 | 0 |
| G+C content (%) | 38.9 | 39.1 | 39.0 | 39.1 | 39.0 | 39.1 |
| Total number of genes | 1,997 | 1,959 | 2,038 | 1,999 | 2,025 | 1,973 |
| Number of protein-coding genes | 1,731 | 1,694 | 1,919 | 1,914 | 1,904 | 1,888 |
| tRNA genes | 68 | 55 | 56 | 67 | 57 | 67 |
| No of ncRNA operons | 4 | 5 | 5 | 6 | 5 | 6 |
| No of CRISPR/Cas | 3 | 4 | 3 | 1 | 2 | 2 |
| Pseudo Genes | 176 | 194 | 115 | 182 | 49 | 180 |
| GenBank accession no. | CP016877 | CP006819 | CP002340 | CP000024 | CP010999 | CP000023 |

S1 Comparison of the General Characteristics of Genome of Sequenced *Streptococcus thermophilus*

S2 EPS gene cluster of *Streptococcus thermophilus* KLDS 3.1003. The predicted functions of each 23 colour-coded ORF are displayed in the lower panel. The size of each ORF in the gene cluster is 23 indicated either as a pentagon (intact) or chevron (truncated)
