# Supplementary figures and images for: Complete Genome Sequence of Streptococcus thermophilus KLDS 3.1003, A Strain with High Antimicrobial Potential against Foodborne and Vaginal Pathogens
Source: Front Microbiol. 2017 Jul 11;8:1238. doi: 10.3389/fmicb.2017.01238 (PMC5504653; doi:10.3389/fmicb.2017.01238)

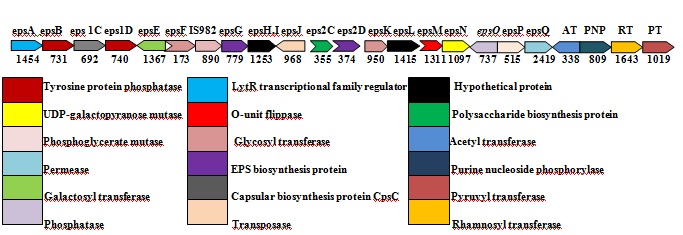

Supplement: Supplementary file 2 [file Image1.jpeg]
